# Oncological outcome and patient satisfaction with skin-sparing mastectomy and immediate breast reconstruction: a prospective observational study

**DOI:** 10.1186/1471-2407-10-171

**Published:** 2010-04-29

**Authors:** Sara Reefy, Neill Patani, Anne Anderson, Gwyne Burgoyne, Hisham Osman, Kefah Mokbel

**Affiliations:** 1 The London Breast Institute, The Princess Grace Hospital, London, UK; 2 St. George's University of London, London, UK

## Abstract

**Background:**

The management of early breast cancer (BC) with skin-sparing mastectomy (SSM) and immediate breast reconstruction (IBR) is not based on level-1 evidence. In this study, the oncological outcome, post-operative morbidity and patients' satisfaction with SSM and IBR using the latissimus dorsi (LD) myocutaneous flap and/or breast prosthesis is evaluated.

**Methods:**

137 SSMs with IBR (10 bilateral) were undertaken in 127 consecutive women, using the LD flap plus implant (n = 85), LD flap alone (n = 1) or implant alone (n = 51), for early BC (n = 130) or prophylaxis (n = 7). Nipple reconstruction was performed in 69 patients, using the trefoil local flap technique (n = 61), nipple sharing (n = 6), skin graft (n = 1) and Monocryl mesh (n = 1). Thirty patients underwent contra-lateral procedures to enhance symmetry, including 19 augmentations and 11 mastopexy/reduction mammoplasties. A linear visual analogue scale was used to assess patient satisfaction with surgical outcome, ranging from 0 (not satisfied) to 10 (most satisfied).

**Results:**

After a median follow-up of 36 months (range = 6-101 months) there were no local recurrences. Overall breast cancer specific survival was 99.2%, 8 patients developed distant disease and 1 died of metastatic BC. There were no cases of partial or total LD flap loss. Morbidities included infection, requiring implant removal in 2 patients and 1 patient developed marginal ischaemia of the skin envelope. Chemotherapy was delayed in 1 patient due to infection. Significant capsule formation, requiring capsulotomy, was observed in 85% of patients who had either post-mastectomy radiotherapy (PMR) or prior radiotherapy (RT) compared with 13% for those who had not received RT. The outcome questionnaire was completed by 82 (64.6%) of 127 patients with a median satisfaction score of 9 (range = 5-10).

**Conclusion:**

SSM with IBR is associated with low morbidity, high levels of patient satisfaction and is oncologically safe for T(is), T1 and T2 tumours without extensive skin involvement.

## Background

The overarching principle guiding surgical management of women with breast cancer (BC) remains oncological safety. The mainstay of satisfactory local control continues to be adequate clearance of the primary tumour and involved axillary lymph nodes. Improvements in our understanding of tumour biology have enabled the risk of loco-regional recurrence (LR) and distant events to be further reduced by adjuvant, or neo-adjuvant, radiotherapy and systemic treatments. In keeping with this, breast-conserving therapy (BCT) has become well established as the treatment of choice for most women with early BC. However, approximately one-third of women still undergo mastectomy, either due to patient preference or in cases where breast conservation is not oncologically or aesthetically compatible with the size or distribution of disease. Despite the relative frequency of mastectomy, guidance from level-1 evidence is lacking in regard to the optimal type of mastectomy which should be performed and the subsequent technique and timing of breast reconstruction.

Skin sparing mastectomy (SSM) involves the en-bloc removal of all glandular tissue including the nipple-areola complex (NAC) and in some cases adjacent biopsy scars and skin overlying superficial tumours. In contrast to conventional mastectomy, there is maximal preservation of the remaining breast skin envelope and infra-mammary fold [1]. SSM can therefore facilitate immediate breast reconstruction (IBR) with autologous tissue and/or prosthetic implants by utilising the native skin envelope to optimise the contour, texture, colour and scarring of the reconstructed breast [1]. This approach combines the ablative and reconstructive components of surgical intervention, offering a single-stage procedure which is likely to be popular with patients in terms of hospital admissions, return to employment and elimination of the post-mastectomy pre-reconstruction period.

The aesthetic advantages of SSM have been tempered to some degree by concerns regarding oncological safety. In comparison with conventional mastectomy, the complete excision of glandular tissue during SSM can be technically more demanding. In addition, there is a perceived increase in the risk of LR attributed to preservation of the skin envelope. Indeed, following conventional mastectomy the most common site for LR is within the skin overlying the chest wall and post-mastectomy radiotherapy (PMR) is recommended for those at high-risk. More than one-third of breast surgeons have been reported to avoid SSM and IBR because of concerns over oncological safety or uncertainty of the benefits or indications [2]. More recently, several studies have contributed to the evidence base supporting the oncological adequacy of SSM in selected early-stage BC, excluding inflammatory BC and tumours with extensive involvement of the skin [1].

In this study the oncological safety, post-operative morbidity and patients' satisfaction with SSM and IBR using the latissimus dorsi (LD) myocutaneous flap and/or breast prosthesis is evaluated in a prospective cohort of women with early-stage BC.

## Methods

The prospective cohort consisted of 127 consecutive women with early-stage BC treated within three independent sector healthcare centres in London. All patients provided informed consent for their inclusion in the prospective observational study, including the use of images within the manuscript. Selection criteria included women with a pre-operative diagnosis (clinical examination, imaging and needle biopsy) of T_is_, T1 and T2 tumours without extensive skin involvement. Only 1 patient included in the study had T3 BC. The principal indication for surgery was BC, however, 7 procedures were undertaken as risk-reducing prophylactic mastectomies (1 BRCA-1 gene carrier, 5 contra-lateral BC). All patients were counselled pre-operatively regarding the ablative and reconstructive options available and surgical recommendations were made on a case-by-case basis following discussion of tumour and patient factors within the context of a multidisciplinary team. Patient related factors included body habitus, size and shape of breasts, co-morbidities, history of smoking and patient preference.

**Figure 1:**
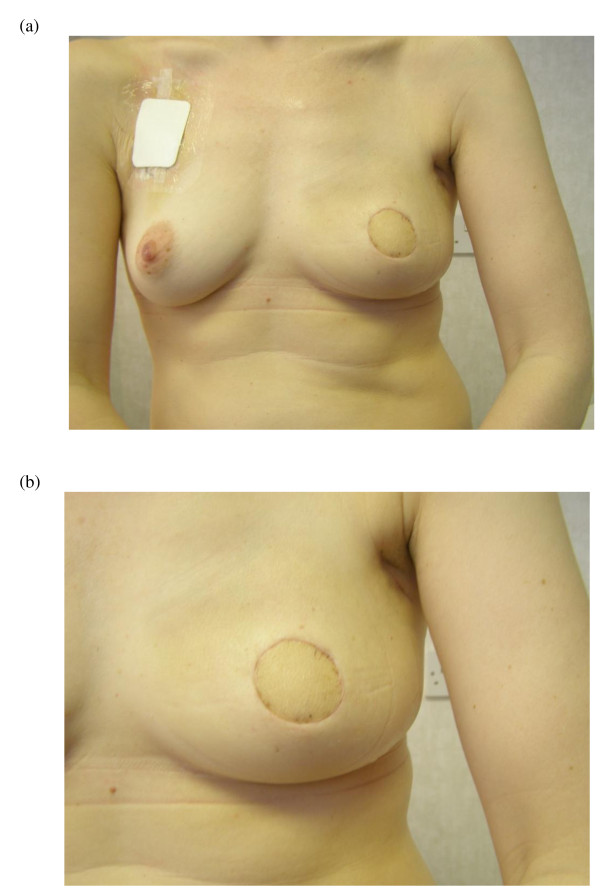
**Skin sparing mastectomy and latissimus dorsi reconstruction**. (a) Left skin sparing mastectomy and latissimus dorsi myocutaneous flap reconstruction in a 45 year old woman prior to nipple reconstruction and tattooing. (b) Magnified view of left side.

**Figure 2:**
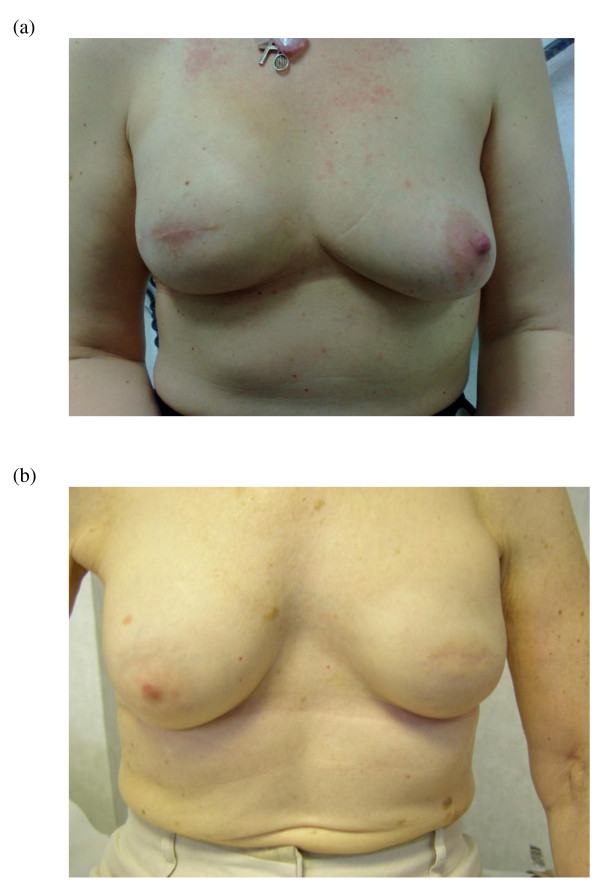
**Skin sparing mastectomy and implant reconstruction**. (a) Right skin sparing mastectomy and implant reconstruction. (b) Left skin sparing mastectomy and implant reconstruction in a 70 year old woman.

**Figure 3:**
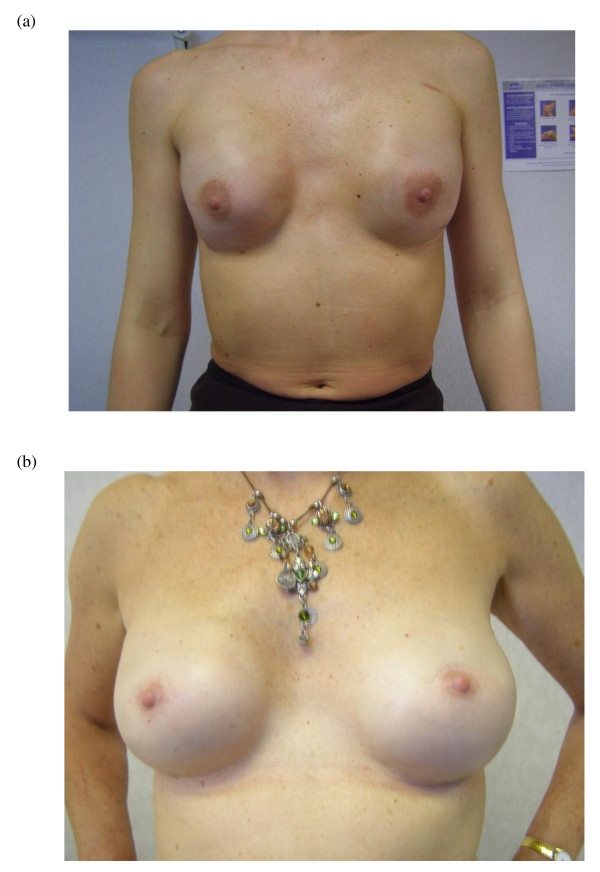
**Nipple sparing mastectomy and implant reconstruction**. (a) Bilateral nipple-sparing mastectomy and implant reconstruction. (b) Bilateral nipple-sparing mastectomy and implant reconstruction for bilateral breast cancer in a 57 year old woman.

**Figure 4:**
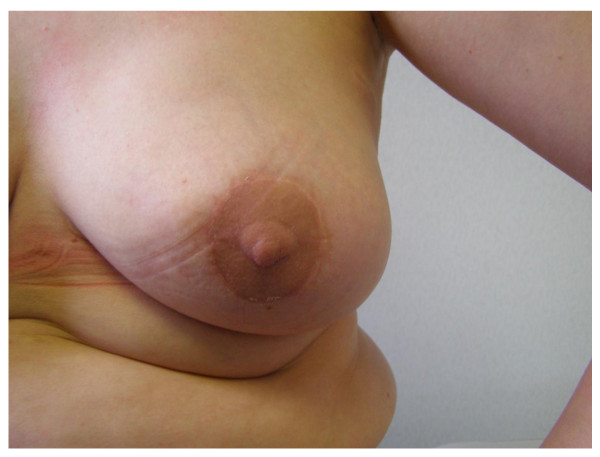
**Nipple reconstruction**. Nipple reconstruction followed by tattooing.

**Figure 5:**
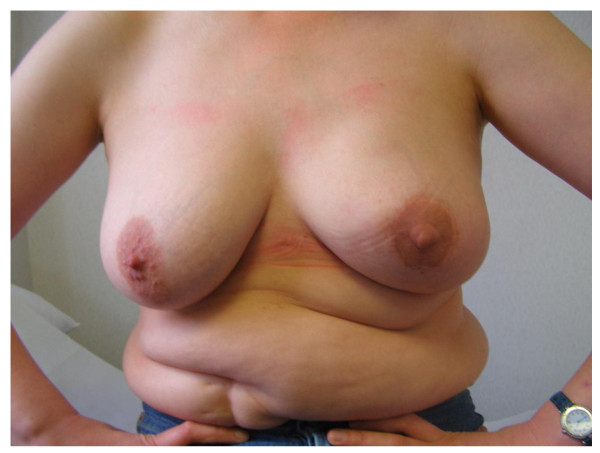
**Skin sparing mastectomy and latissimus dorsi flap with nipple reconstruction**. Left skin sparing mastectomy and latissimus dorsi myocutaneous flap reconstruction, followed by nipple reconstruction and tattooing.

**Figure 6:**
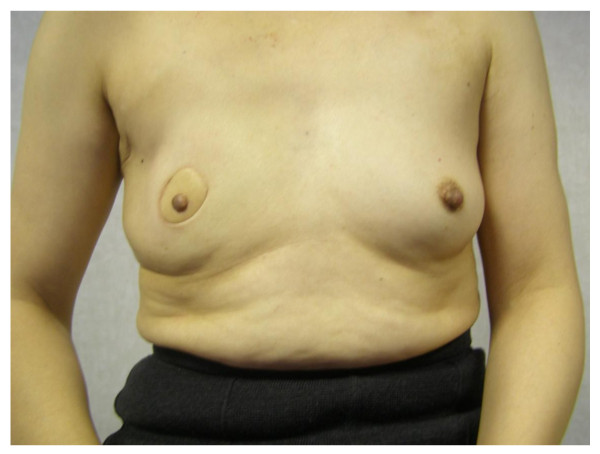
**Skin sparing mastectomy and extended latissimus dorsi flap with nipple reconstruction**. Right SSM and IBR using an extended LD flap without an implant in a 56 year old woman. She subsequently had nipple reconstruction using a free graft from the left nipple.

Surgical procedures were performed by the same surgeon (K.M.) between 2001 and 2008. All 127 women underwent SSM and IBR, with 117 unilateral and 10 bilateral procedures, providing a total of 137 cases. Nipple-sparing mastectomy (NSM) and IBR were undertaken in 10 cases (6 patients). IBR employed the LD pedicle-flap and implant (n = 85, including 1 bilateral case), LD flap alone (n = 1) or implant only (n = 51, including 9 bilateral cases), Figures 1, 2, 3, 4, 5 and 6. Information regarding reconstructive options was provided to all patients pre-operatively. Multidisciplinary recommendations were subsequently made on an individual basis, reflecting the aforementioned tumour and patient factors. In particular, LD pedicle-flap and implant reconstructions were undertaken in those women for whom an implant only reconstruction would not provide satisfactory cosmesis. In some cases, the intention was not simply to match the reconstructed breast as closely as possible to the contra-lateral breast, but rather to provide the patient with a more satisfactory 'matching pair' of breasts by enhancement or reduction of the native breast. Hence, thirty patients underwent contra-lateral surgery in order to optimize symmetry and cosmesis. These procedures consisted of 19 augmentations and 11 mastopexy/reduction mammoplasties. The initial implant used in all cases was a tissue expander, in order to optimise the size and shape of the reconstructed breast. This was subsequently replaced in most patients, with an anatomically profiled bio-dimensional cohesive silicon implant, at the same time as nipple reconstruction or contra-lateral adjustment thus adding no unnecessary surgical episodes. Nipple reconstruction was performed in 69 patients using the trefoil local flap technique (n = 61), nipple sharing (n = 6), skin graft (n = 1) and Monocryl mesh (n = 1), Figures 4 and 5.

Patients at high risk of requiring PMR were encouraged to opt for SSM and IBR using an implant alone (tissue expander). Following PMR, these patients were reviewed to assess the need for delayed conversion to an autologous flap with exchange of the tissue expander for definitive prosthesis, particularly if the aesthetic outcome was significantly compromised. We adopted this 'immediate-delayed' strategy in 11 cases. All patients underwent clinical examination on a six monthly basis and annual surveillance mammography. The patient's satisfaction with the outcome of surgery was assessed using a linear visual analogue scale, ranging from 0 (not satisfied) to 10 (most satisfied), through a postal questionnaire, Additional File 1. The mean time from treatment to issue of the questionnaire was approximately 18 months.

### Surgical Considerations

In the majority of cases SSM was performed through a peri-areolar incision. In a small number of women the peri-areolar incision provided inadequate access to remove all glandular tissue and additional short horizontal incisions at the 3 and 9 o'clock positions were guided by breast size, profile and skin laxity. The infra-mammary fold was preserved in all cases. In patients who underwent NSM, the tumour was always more than 2.5 cm from the NAC and an intra-operative frozen-section protocol was followed to ensure that the retro-areola tissue was tumour free. All mastectomy specimens were sent for definitive histopathological analysis. All patients had pre-operative axillary ultrasound +/- fine needle aspiration cytology (FNAC) and those with negative axillae underwent sentinel lymph node biopsy (SLNB) using blue dye and/or radioactive isotope tracer, usually through a separate short axillary incision. The SLNB protocol evolved during the course of the study; between 2001-2004 the technique was based on a blue-dye guided sample aiming to retrieve four nodes, whereas between 2005-2009 the dual localization technique was utilised. Immediate axillary node clearance was performed if intra-operative frozen section analysis of the SLNB showed malignancy. Subsequent replacement of the tissue expander with definitive implant prosthesis was performed through short infra-mammary incisions. The prosthesis was placed in a sub-muscular pocket in all cases. Negative suction drains were used in all patients at the LD donor site and adjacent to the prosthetic implants. All patients received prophylactic antibiotics and low molecular weight heparin.

## Results

The median patients' age was 47 years (range = 27-72). Histopathological analysis of mastectomy specimens showed pure DCIS in 25 cases and invasive carcinoma (+/- DCIS) in 105 cases, Table 1. One patient carrying BRCA-1 gene mutation had bilateral prophylactic mastectomies with normal histology. The median tumour size was 28 mm (range 1 mm - 100 mm) and in all cases the superficial surgical margins were clear with no cancer cells at the surgical margins on microscopic examination. In two patients with extensive multi-centric disease the tumour extended to the medial surgical margin (focal DCIS only) and one of these patients received PMR. Lymph nodes were found to be involved in 45 patients, 41 of whom had macro-metastasis and a further 4 patients had evidence of micro-metastatic spread within the sentinel node. In total, 23 patients had axillary clearance at the first operation. Of these, 20 women were suspected to be node positive pre-operatively by axillary ultrasound and FNAC and this was confirmed on final histopathological analysis. The other 3 patients were found to be node negative on definitive analysis, resulting from the fact that SLNB was not available during the first month of the study. The remainder of the cohort had intra-operative SLNB to stage the axilla, proceeding to immediate axillary clearance if indicated by positive frozen section analysis. We observed no cases which were intra-operatively negative by frozen section and subsequently positive on final histopathological analysis, hence no patients returned to theatre for completion clearance. Adjuvant chemotherapy was required by 38 patients. Prior RT had been received by 6 patients, who had previously undergone BCT for cancer, comprising wide-local excision and adjuvant RT. They subsequently developed recurrence and were managed with SSM & IBR. In addition, 21 women underwent PMR (LD flap and implant reconstruction = 10, implant only reconstruction = 11).

**Table 1:** The histological findings in SSM specimens (n = 137) classified according to the TNM stage at diagnosis.

TNM stage	No. of cases	Local Recurrence	Distant Recurrence
T_is_	23	0	0
TisN1	2	0	0
T1N0 M0	47	0	2
T1N1 M0	16	0	0
T2N0 M0	15	0	2 (one mortality)
T2N1 M0	26	0	4
T3N1 M0	1	0	0
Normal/Benign	7	0	0
**Total**	**137**	0	8

There was no LR after a median follow-up of 36 months (range = 6-101 months). Overall survival was 99.2%. Eight patients developed distant disease and one patient died of metastatic breast cancer. One other patient died of lung cancer. In our series, we observed no cases of partial or total LD flap loss. Morbidities included infection, requiring implant removal in 2 patients and 1 patient (smoker) developed marginal ischaemia of the skin envelope which was treated conservatively. Six patients had previous RT and none of them developed wound complications. Negative suction drains were used in all patients with a median period of 5 days for the LD donor-site and 3 days for drains adjacent to prosthetic implants. All patients undergoing LD flap reconstruction developed donor-site seromas which required percutaneous drainage in the outpatient setting; the median inpatient stay was 5 days. Chemotherapy was delayed by 2 weeks in 1 patient due to infection. In 2 patients excessive oozing from the LD harvest site resulted in a decrease in haemoglobin sufficient to necessitate blood transfusion (<8 g/dL), however this was self limiting in both cases and no re-operation was needed. There were no admissions to the intensive care unit. Significant capsule formation was observed in approximately 85% (23/27) of patients who had either prior RT or PMR, which was substantially greater than the 13% (13/100) for those patients who had not received any RT. Capsule formation was corrected with capsulotomy at the time of exchanging the tissue expander for definitive prosthesis. However, one patient required a second capsulotomy several months after placement of the definitive prosthesis. Eighty-two (64.6%) of 127 patients completed the satisfaction survey with a median score of 9 (range = 5-10). There was no significant difference in satisfaction scores between patients undergoing IBR using LD flap and implant reconstruction (mean = 9.3, median = 10) and those undergoing implant only reconstruction (mean = 9, median = 10).

## Discussion

Over the last two decades there has been a paradigm shift in the surgical management of BC away from radical and ablative surgery towards a more tailored and conservative approach. Improvements in our understanding of the natural history and tumour biology of BC, alongside the advent of evidence-based adjuvant local and systemic therapies, have concluded the era where oncological safety and aesthetic outcome were perceived to be mutually exclusive. BCT now represents the standard of care for women with early-stage BC. In keeping with this evolution in surgical practice, SSM can be considered part of the rational progression away from conventional mastectomy. Several studies have recently evaluated SSM for BC and found the incidence of LR to comparable to conventional mastectomy. In a 15 year retrospective series of women with stage 0-2 IBC, 225 patients undergoing SSM and IBR were compared to 1022 patients treated by conventional mastectomy. After an average follow-up of 49 months, there was found to be no significance difference in LR [3]. After an average follow-up of 51 months, Meretoja et al. reported only 4 LRs within the native breast skin of 146 women with stage 0-2 IBC. Following surgical and oncological treatment none of these patients developed new recurrences after a mean of 35 months, suggesting that not all LRs are associated with disseminated disease [4]. In another series of 105 patients undergoing SSM and IBR, followed-up for an average of 48 months, only one case of LR was identified [5]. In their retrospective study with an average follow-up of 58 months, Vaughan et al. found 11(5.3%) cases of LR in 210 SSMs with IBR, 9 of which developed in the quadrant of the corresponding primary tumour [6]. In our series, we observed no cases of LR after a median follow up of approximately 3 years. Although longer follow up is required, the present study adds to the growing body of evidence that SSM is oncologically safe for early stage breast cancer (Tis, T1 and T2 tumours) without extensive skin involvement [1,7]. Evidence in support of the oncological adequacy of SSM in selected cases continues to increase in quantity, quality and maturity of the data. A recent postal questionnaire survey of 370 Californian surgeons suggests that attitudes and practices may also be changing, with 90% of respondents satisfied with oncological adequacy and 70% in agreement with regard to superior aesthetic outcome [8]. Our series also included one patient with a T3 tumour, although the utility of SSM and IBR in this subgroup has been controversial, particularly with regard to the risk of LR within the preserved skin envelope [9]. One study of 25 patients with locally advanced breast cancer (stage IIB/stage III), found an LR rate of 4% after a median follow-up of 49 months [10]. However, a retrospective study of 207 women undergoing SSM and IBR, with a mean follow-up of 70 months, 5.8% of patients with stage 0-2 disease developed LR, compared to 31% of women with stage 3 BC [11]. Recognised risk factors for LR after SSM and IBR include: tumour size, stage, poor differentiation and lymph node involvement [12]. Uriburu et al. [13] also recommend surgical resection of any needle biopsy tracts at the time of SSM to reduce the risk of biopsy site LR.

SSM can facilitate IBR by the advantages in contour, colour, texture and scarring associated with preservation of the native skin envelope. This approach can also reduce the need for contra-lateral adjustment in order to achieve symmetry [2,14]. In their study of 112 patients who underwent SSM and IBR, Gerber et al [15] recently reported a 91% patient satisfaction rate with aesthetic outcome. The combination of ablative and reconstructive procedures, offers a single-stage intervention which is popular with patients in terms of hospital stay, return to work and elimination of the post-mastectomy pre-reconstruction period. In the present study, approximately two-thirds of all patients completed the questionnaire and the median patient satisfaction score was 9/10. This study also demonstrates that IBR using an LD flap and/or implant is an acceptable option for most patients without the need for a more complex reconstruction using free tissue transfer and micro-vascular surgery. The fact that patients' satisfaction with the surgical and aesthetic outcome was only assessed subjectively using an analogue score represents an important limitation to our study. A larger sample size would be required to detect small differences between particular patient subgroups. Furthermore, patients' satisfaction may change over time, particularly in women with implant-based reconstructions, and we shall continue to monitor this in the long-term. Follow up is also required to assess the number of procedures which may be necessary to deal to implant complications.

SSM and IBR in our series carried a low morbidity. Skin flap necrosis is well recognised and may result from compromised blood supply secondary to excessive undermining and thinning of the flaps. We observed only one case of marginal ischemia of the skin envelope (1%) in a current smoker. This complication has been estimated to occur in 11% of SSM and non-SSM cases [16]. The low incidence in our study may be attributed to the extreme care taken during dissection in order to accurately identify and follow the superficial fascial plane between the subcutaneous fat and the mammary tissue. Furthermore the low rate of infection leading to implant loss (2%) in our cohort can be attributed to the routine use of prophylactic antibiotics. In the present study, a large proportion of patients who underwent prior RT or PMR developed capsular fibrosis and shrinkage, resulting in discomfort and deformity. However, this complication was effectively managed by capsulotomy at the time of exchanging the tissue expander for definitive prosthesis. Surgical complications can compromise the aesthetic outcome of SSM and IBR and this has been associated with a reduction in patient satisfaction [17].

### Skin-Sparing Mastectomy and Ductal Carcinoma In-Situ

Mastectomy can also be indicated in several non-invasive conditions. DCIS may necessitate mastectomy when the lesions are extensive, multi-centric or recurrent, however, sometimes patients request to be managed in this way. Mastectomy for DCIS is associated with cure rates in excess of 98% [18]. In our study SSM and IBR was undertaken for pure DCIS in 25 cases and there were no cases of LR. In a study by Rubio et al. [19] where 95 patients underwent SSM and IBR for DCIS, 98% were alive and disease free after a median follow-up of 3.7 years. In 35 cases, intra-operative specimen radiography and histological examination of serial sections were used to confirm clearance of the margins and none of these developed LR. The overall LR rate was 3%. The series by Carlson et al. [20] included 175 patients with DCIS and identified only 1 LR after 65 months of follow-up. Similarly, Slavin et al. [21] reported no LR in a cohort of 26 patients with DCIS after 45 months of follow-up. Studies with longer follow-up also provide evidence for oncological safety, Spiegel and Butler [22] found no LR after 9.8 years. SSM and IBR is particularly attractive for women with DCIS in view of the fact that PMR is not given to the reconstructed breast and the risk of LR is very low.

### Radiotherapy & Skin-Sparing Mastectomy

Most women who undergo SSM and IBR for early-stage BC will not require PMR. However, PMR has been shown to reduce LR and improve survival for patients with four or more involved regional lymph nodes or tumours >5 cm [23]. Complication rates of 5-16% have been reported with PMR following autologous breast reconstruction [2,16]. In a study of 377 implant reconstructions, morbidities including pain and capsular contracture were reported following PMR [24]. Indeed, patient satisfaction has been found to be lower in those who undergo RT (67% vs. 88%) [25]. Significant capsule formation was observed in the majority of our patients who had PMR, however, this was effectively treated with capsulotomy at the time of implant replacement and we did not observe a significant reduction in satisfaction scores. The management of women who are likely to need PMR continues to be controversial and some surgeons advocate a conventional mastectomy and delayed reconstruction. More recently, it has been suggested that the benefits of SSM can be preserved in this subgroup using an 'immediate-delayed' reconstructive technique [2]. A temporary sub-pectoral tissue expander can be placed at the time of SSM. Following PMR, delayed reconstruction can be performed by replacing the expander with a myocutaneous flap and/or implant. This approach avoids the potential radiotherapy delivery problems and cosmetic disadvantages associated with IBR followed by PMR [26]. We employed this strategy in 11 patients who required PMR in our series. Alternatively, prior to mastectomy, radiological tumour size, analysis of core-biopsies and SNLB could be used to assess the likelihood of PMR, thereby facilitating patient selection. Randomised controlled trials are now required to compare the oncological and aesthetic outcomes for those women who require PMR and are treated by SSM and IBR, conventional mastectomy with delayed reconstruction or immediate-delayed reconstruction.

### Nipple-Sparing Mastectomy & Immediate Breast Reconstruction

Several variations of the standard SSM have recently been reported, including the NSM. Preservation of the NAC offers aesthetic advantages and eliminates the need for nipple reconstruction associated with standard SSM. Oncological concerns regarding the risk of occult NAC involvement have been assuaged to some extent by several recent studies [2,27-29]. Voltura et al [30] recently reported outcomes for 34 NSMs undertaken for cancer (24 BC and 10 DCIS). In each case histological analysis of the sub-areolar tissue was performed and only 2 cases (5.9%) required subsequent NAC removal. These results are in keeping with a similar study reported by Wijayanayagam et al [31]. The risk of NAC involvement has been corroborated in a larger retrospective series of 286 SSMs, 16 (5.6%) were found to contain tumour in the NAC [27]. If multi-centric and sub-areolar tumours were excluded, the NAC was only involved in 3% of cases. Gerber et al. [32] performed 112 SSMs in women whose breast cancer was more than 2 cm from the NAC and used frozen sections of the retro-areola tissue to determine NAC preservation. The cosmetic results were independently evaluated as excellent or good in 91% and were significantly better after NAC preservation. Only one LR occurred in the NAC preservation group. Frozen section analysis of the retro-areola tissue has been found to be 90.9% sensitive and 98.5% specific [33]. Using this approach, Benediktsson and Perbeck have recently reported LR rates of 28.4%, falling to 8.5% in those receiving PMR, in series of 216 women undergoing NSM after a median follow-up of 13 years. These are comparable to conventional mastectomy. Overall, 91% of all preserved NACs were present at the end of the study. Furthermore, LR was not found to be associated with a reduced overall survival. Another series of 140 mastectomies found tumour size and nodal positivity to be risk factors for NAC involvement [28]. Furthermore, the primary tumour was situated within 2.5 cm of the areola in all cases in which the NAC was positive. A retrospective study involving 217 mastectomy specimens by Simmons et al. [29] reported NAC tumour involvement in 23 cases (10.6%). It was also found that only 6.7% of small tumours with up to two positive lymph nodes had NAC involvement. Therefore, it would appear oncologically safe to perform SSM with NAC preservation, provided the tumour is not close to the nipple and a frozen section protocol is followed.

Another variation of the standard SSM involves removal of the nipple with areola preservation, termed the areola-sparing mastectomy (ASM). The concept is supported by Simmons et al. [29] who found that areola involvement was only identified in 2 of the 23 positive NACs. ASM can therefore maintain cosmesis and would only require a subsequent nipple reconstruction, if requested by the patient. Simmons et al. [34] have reported a series of 17 cases with only a single complication (wound infection) over a 20-month period.

## Conclusion

SSM and IBR is oncologically adequate in selected patients with T_is_, T1 and T2 tumours in the absence of extensive skin involvement. The approach is associated with high levels of patient satisfaction and low morbidity. Randomised controlled trials are required to compare the oncological and aesthetic outcomes for those women who require PMR and are treated by SSM and IBR, conventional mastectomy with delayed reconstruction or immediate-delayed reconstruction.

## Conflicts of interests

The authors declare that they have no competing interests.

## Authors' contributions

SR participated in literature review, data interpretation and manuscript writing. NP carried out literature review, data interpretation, manuscript writing & editing. AA undertook data collection. GB undertook data collection. HO participated in literature review. KM was the principal investigator, lead clinician and operating Surgeon, also responsible for study design/coordination and patient recruitment. All authors read and approved the final manuscript.

## Pre-publication history

The pre-publication history for this paper can be accessed here:

http://www.biomedcentral.com/1471-2407/10/171/prepub

## Supplementary Material

Additional file 1 Outcome questionnaire employed in study. Click here for file
