# Supplementary figures and images for: Oncological outcome and patient satisfaction with skin-sparing mastectomy and immediate breast reconstruction: a prospective observational study
Source: BMC Cancer. 2010 Apr 29;10:171. doi: 10.1186/1471-2407-10-171 (PMC2873394; doi:10.1186/1471-2407-10-171)

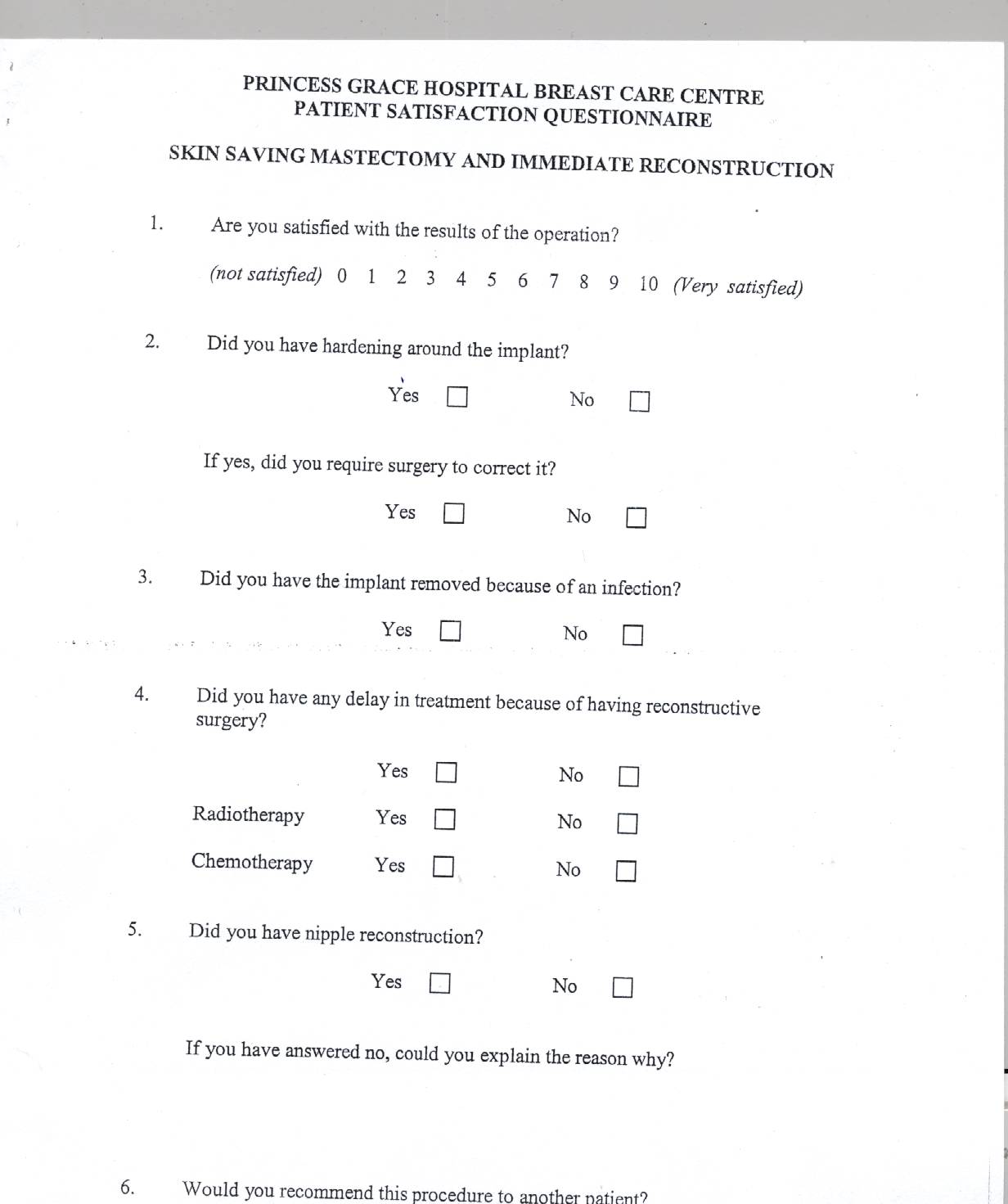

Supplement: Additional file 1 — Outcome questionnaire employed in study. [file 1471-2407-10-171-S1.DOC]
